# Supplementary material for: A Structural Model for Bax∆2-Mediated Activation of Caspase 8-Dependent Apoptosis
Source: Int J Mol Sci. 2020 Jul 31;21(15):5476. doi: 10.3390/ijms21155476 (PMC7432750; doi:10.3390/ijms21155476)
Supplement: Supplementary file 1 [file ijms-21-05476-s001.pdf]

Fig. S1

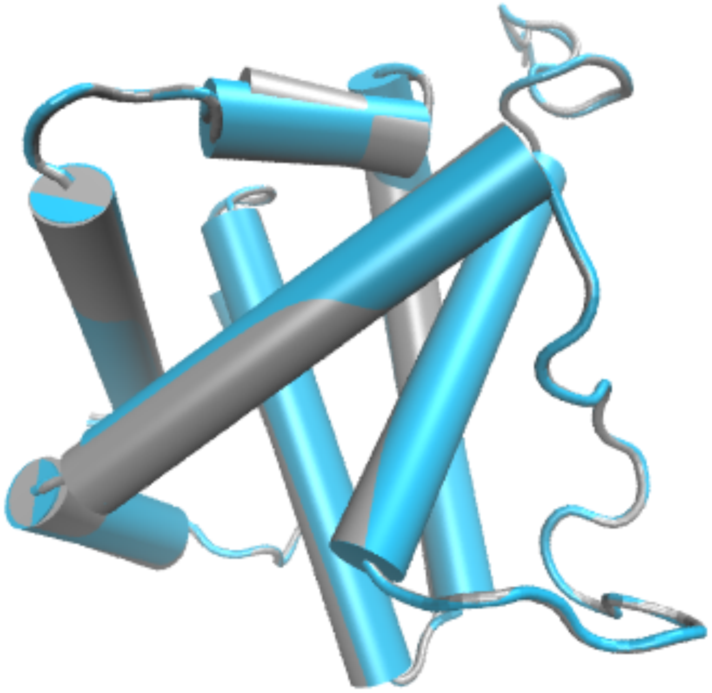

RMSD: 0.317

Fig. S2

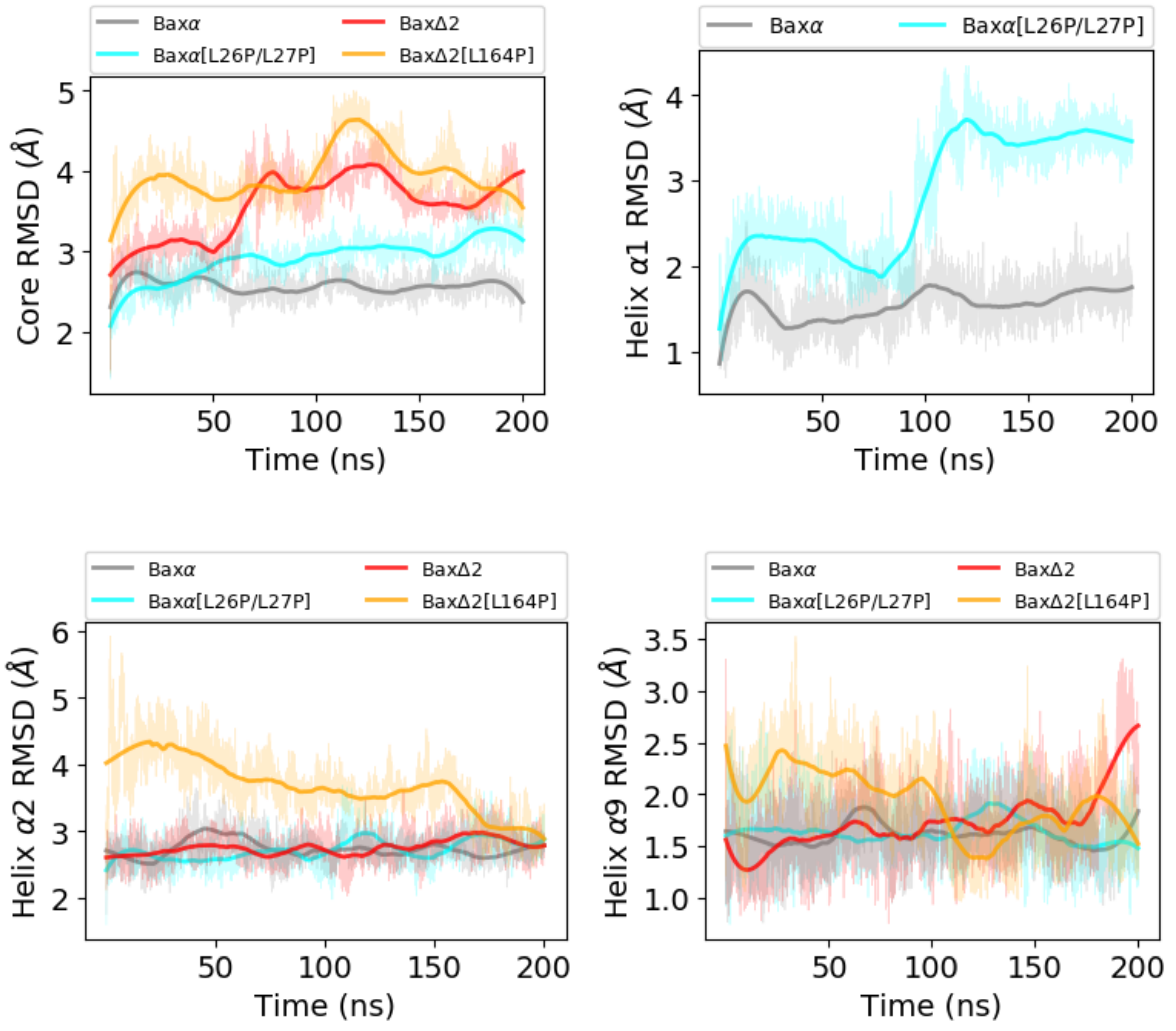
Baxα
BaxΔ2
Baxα[L26P/L27P]
BaxΔ2[L164P]
Core RMSD (Å)
Time (ns)
Baxα
Baxα[L26P/L27P]
Helix α1 RMSD (Å)
Time (ns)
Baxα
BaxΔ2
Baxα[L26P/L27P]
BaxΔ2[L164P]
Helix α2 RMSD (Å)
Time (ns)
Baxα
BaxΔ2
Baxα[L26P/L27P]
BaxΔ2[L164P]
Helix α9 RMSD (Å)
Time (ns)

Fig. S3

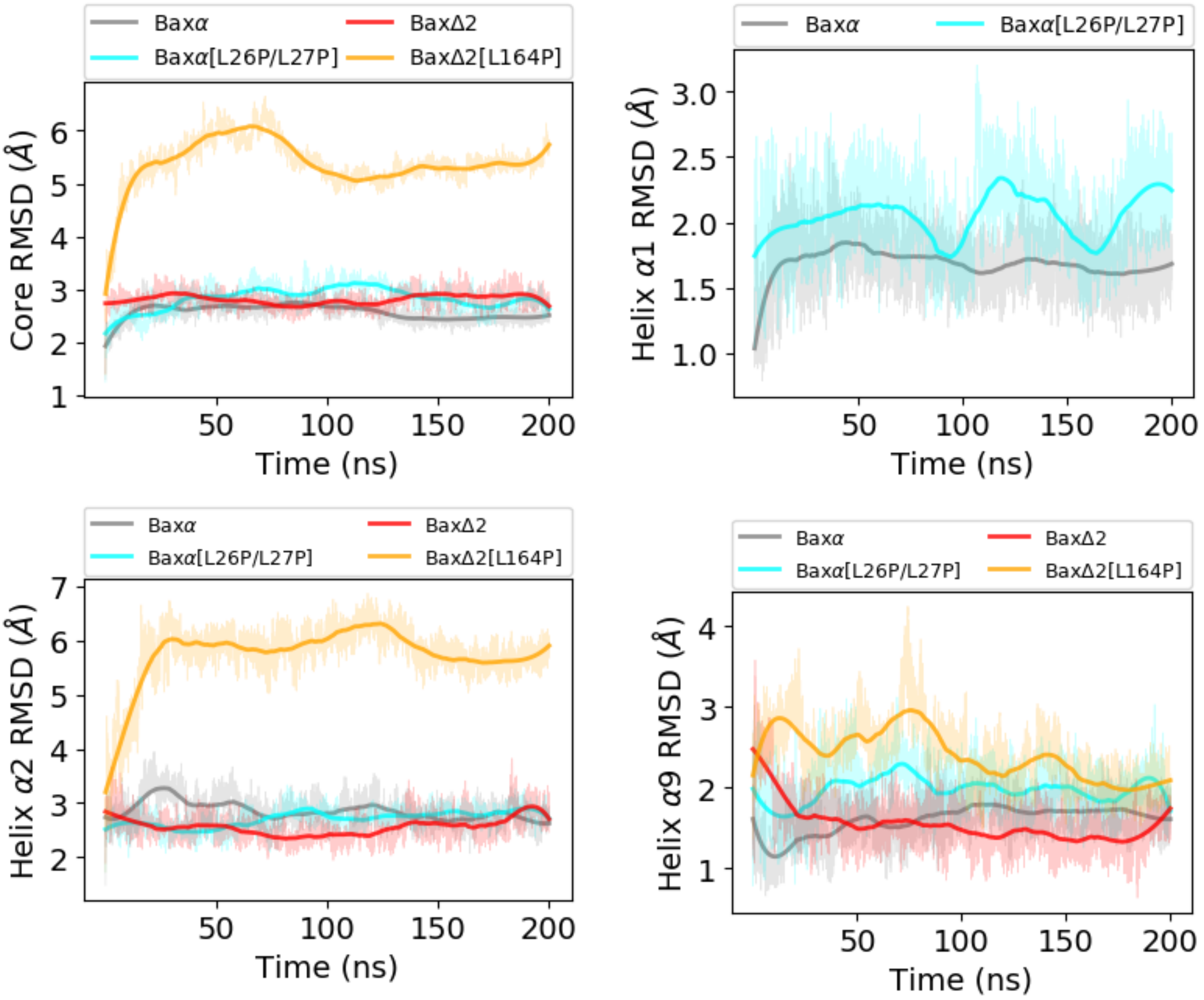

Baxα
BaxΔ2
Baxα[L26P/L27P]
BaxΔ2[L164P]
Core RMSD (Å)
Time (ns)
Baxα
Baxα[L26P/L27P]
Helix α1 RMSD (Å)
Time (ns)
Baxα
BaxΔ2
Baxα[L26P/L27P]
BaxΔ2[L164P]
Helix α2 RMSD (Å)
Time (ns)
Baxα
BaxΔ2
Baxα[L26P/L27P]
BaxΔ2[L164P]
Helix α9 RMSD (Å)
Time (ns)

Fig. S4

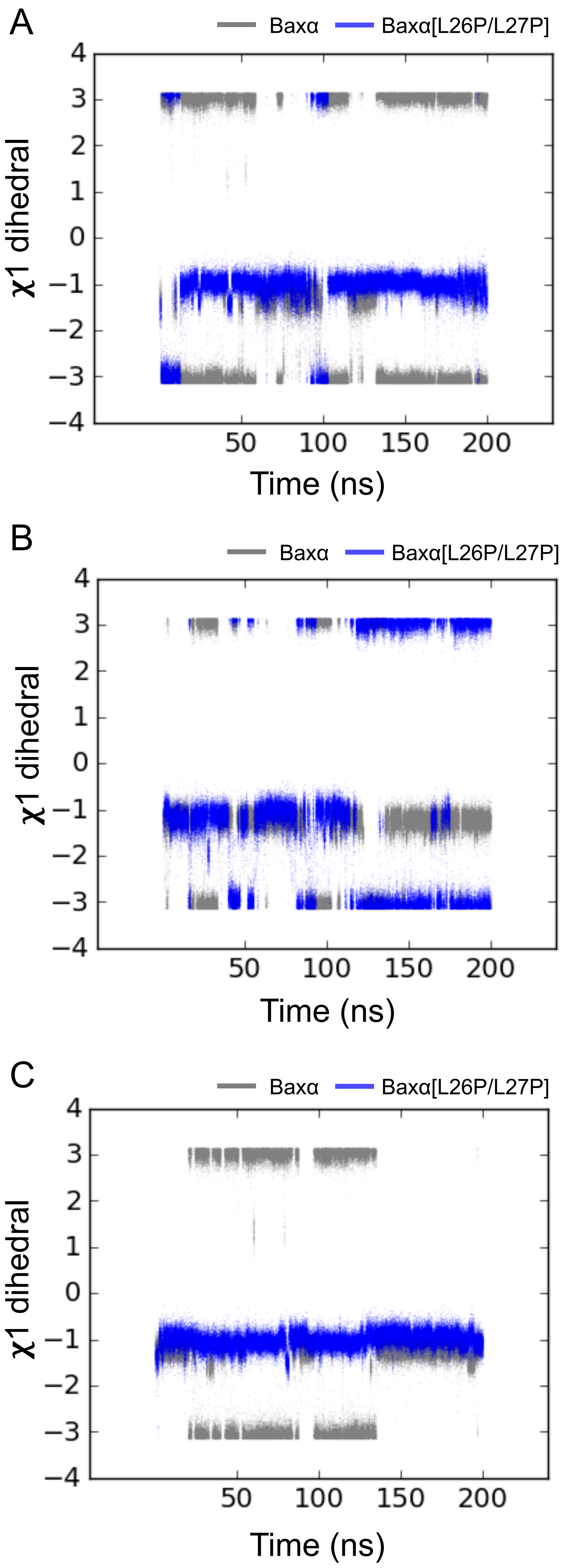
A
Baxα
Baxα[L26P/L27P]
χ1 dihedral
Time (ns)
B
Baxα
Baxα[L26P/L27P]
χ1 dihedral
Time (ns)
C
Baxα
Baxα[L26P/L27P]
χ1 dihedral
Time (ns)

Fig. S5

A

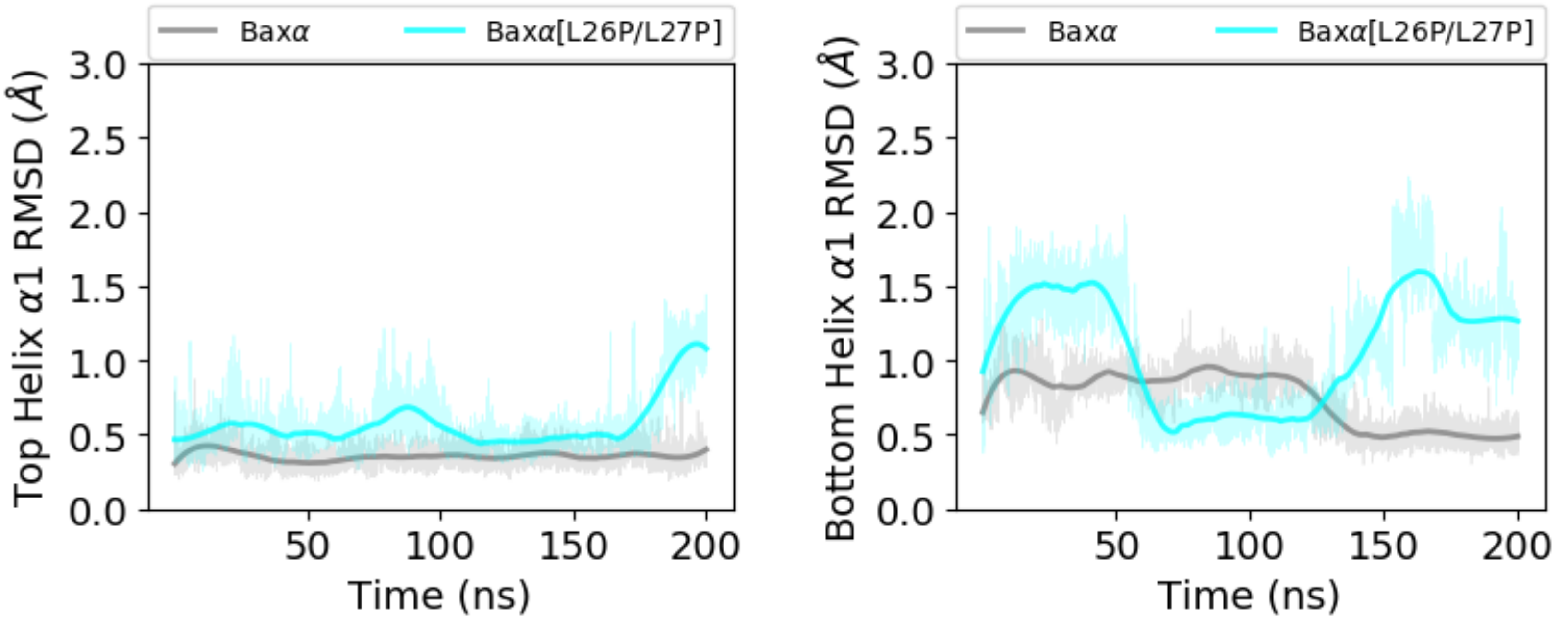

Baxα
Baxα[L26P/L27P]
Top Helix α1 RMSD (Å)
Bottom Helix α1 RMSD (Å)
Time (ns)

B

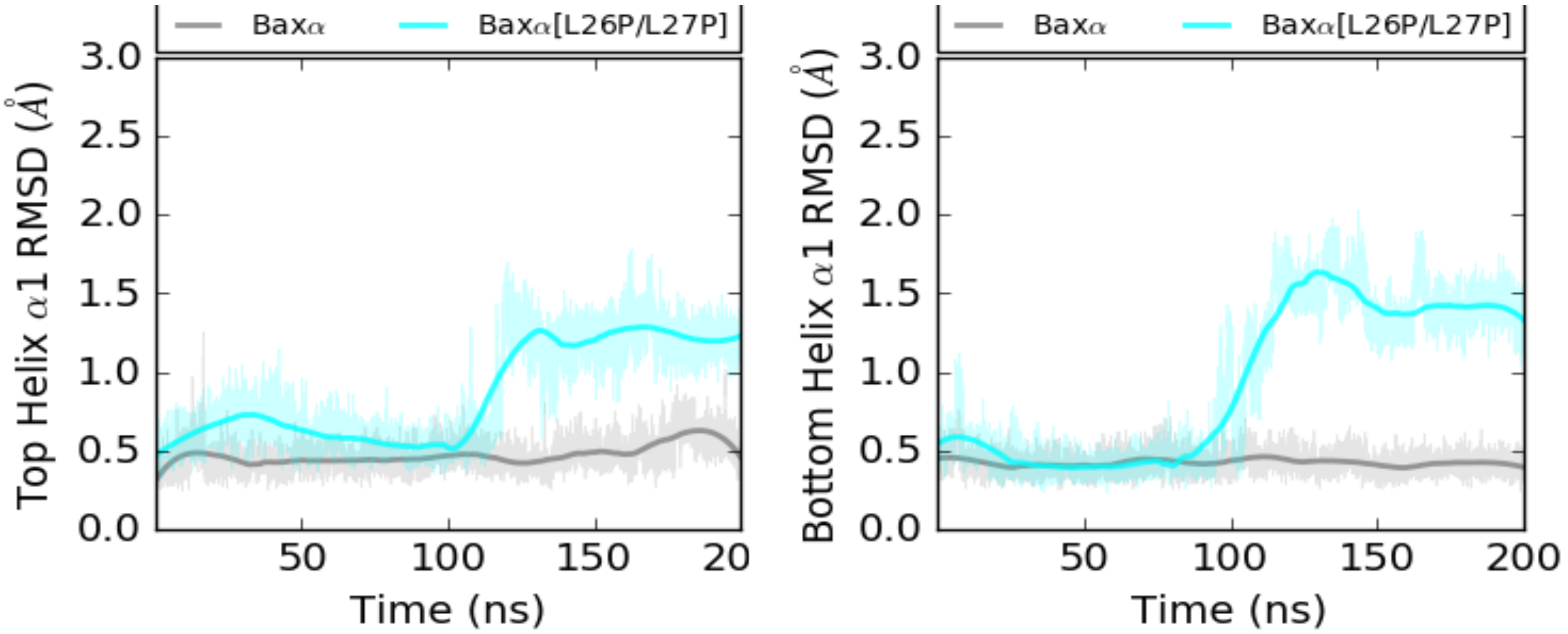

Baxα
Baxα[L26P/L27P]
Top Helix α1 RMSD (Å)
Bottom Helix α1 RMSD (Å)
Time (ns)

C

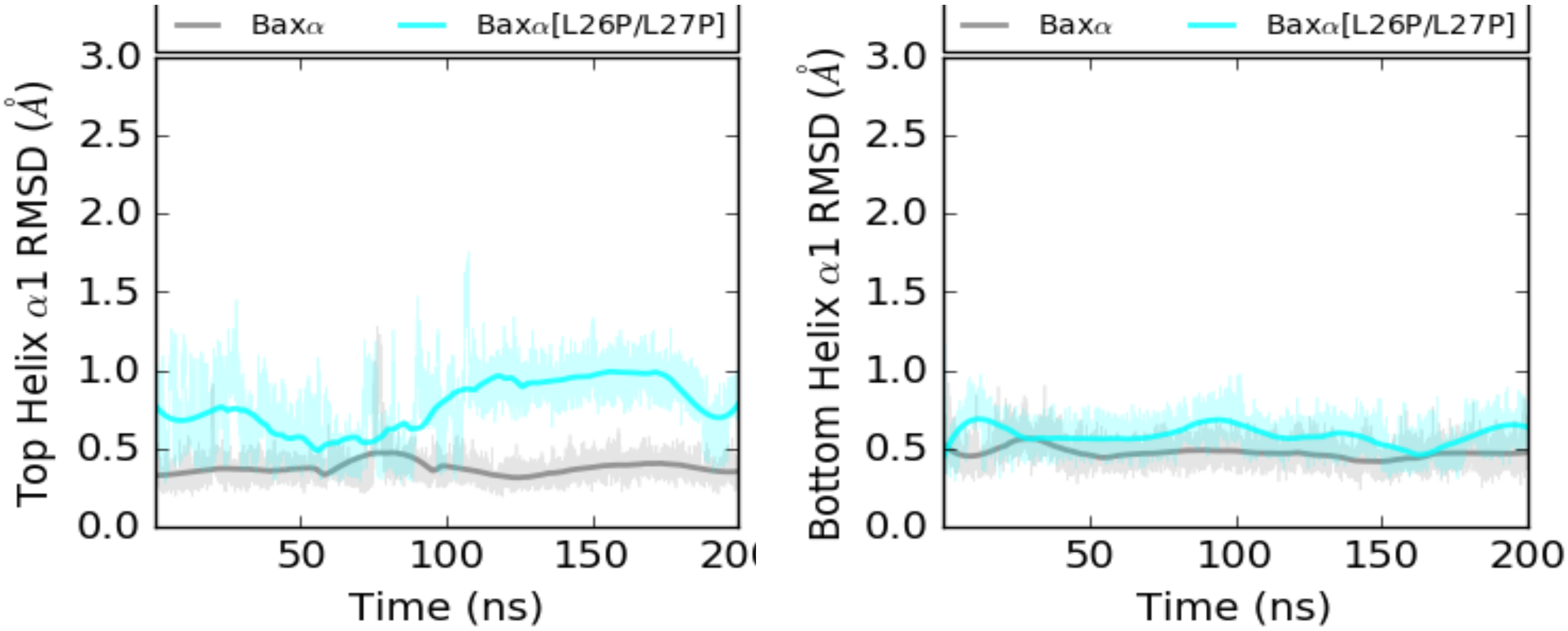

Baxα
Baxα[L26P/L27P]
Top Helix α1 RMSD (Å)
Bottom Helix α1 RMSD (Å)
Time (ns)

Fig. S6

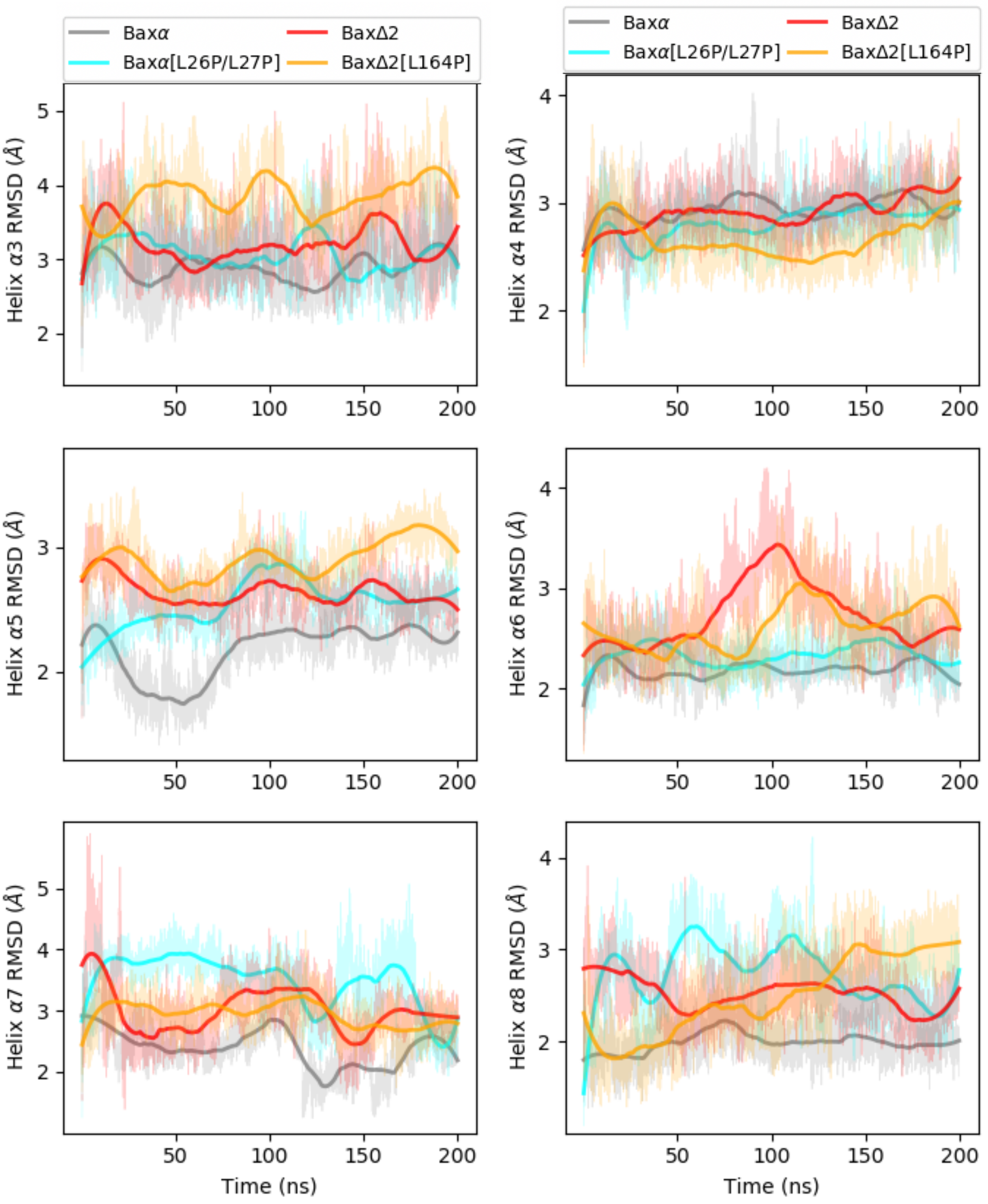
Baxα
BaxΔ2
Baxα[L26P/L27P]
BaxΔ2[L164P]
Helix α3 RMSD (Å)
Helix α4 RMSD (Å)
Helix α5 RMSD (Å)
Helix α6 RMSD (Å)
Helix α7 RMSD (Å)
Helix α8 RMSD (Å)
Time (ns)
Time (ns)

Fig. S7

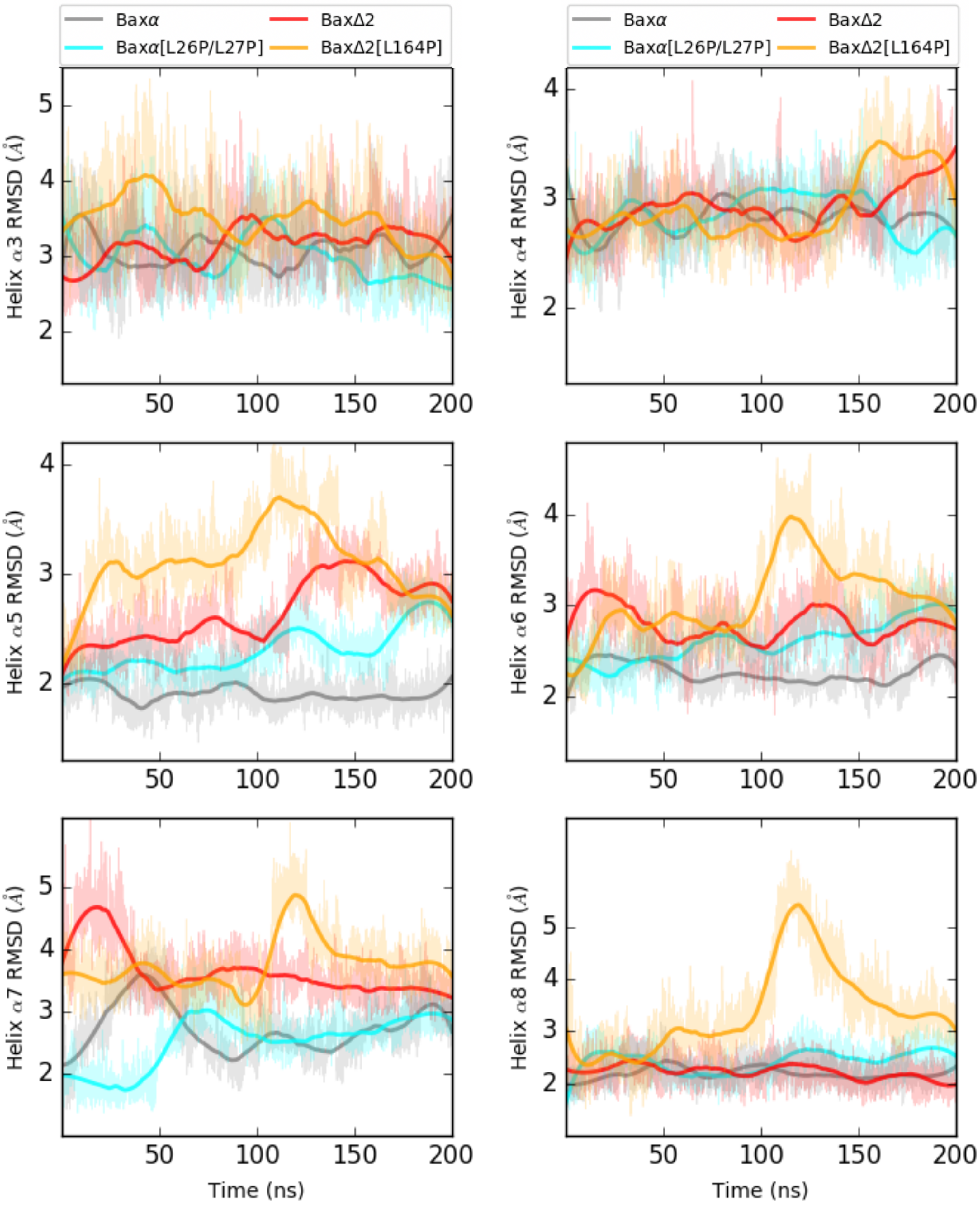

Baxα
BaxΔ2
Baxα[L26P/L27P]
BaxΔ2[L164P]
Helix α3 RMSD (Å)
Helix α4 RMSD (Å)
Helix α5 RMSD (Å)
Helix α6 RMSD (Å)
Helix α7 RMSD (Å)
Helix α8 RMSD (Å)
Time (ns)
Time (ns)

Fig. S8

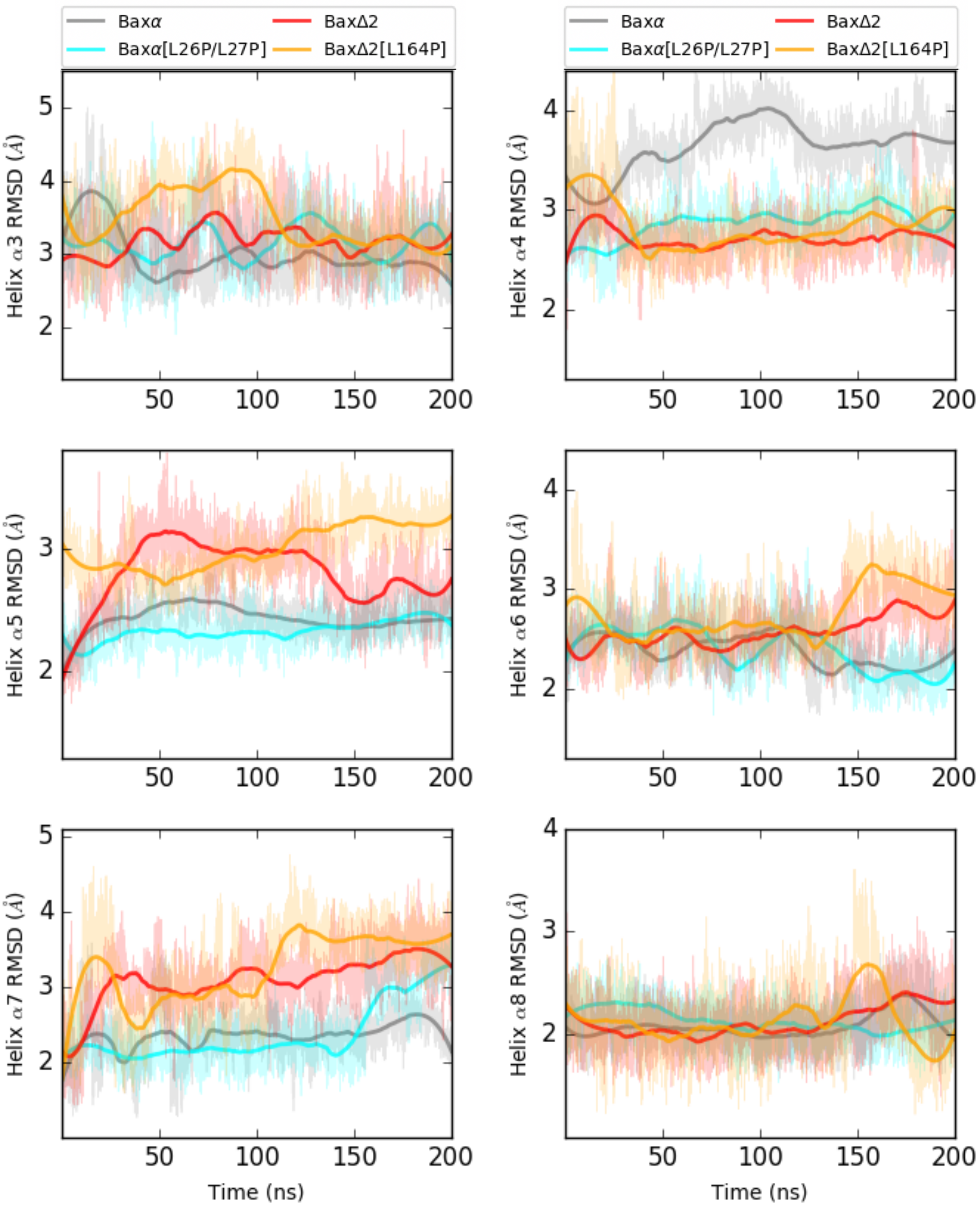

Baxα
BaxΔ2
Baxα[L26P/L27P]
BaxΔ2[L164P]
Helix α3 RMSD (Å)
Helix α4 RMSD (Å)
Helix α5 RMSD (Å)
Helix α6 RMSD (Å)
Helix α7 RMSD (Å)
Helix α8 RMSD (Å)
Time (ns)
Time (ns)

Fig. S9

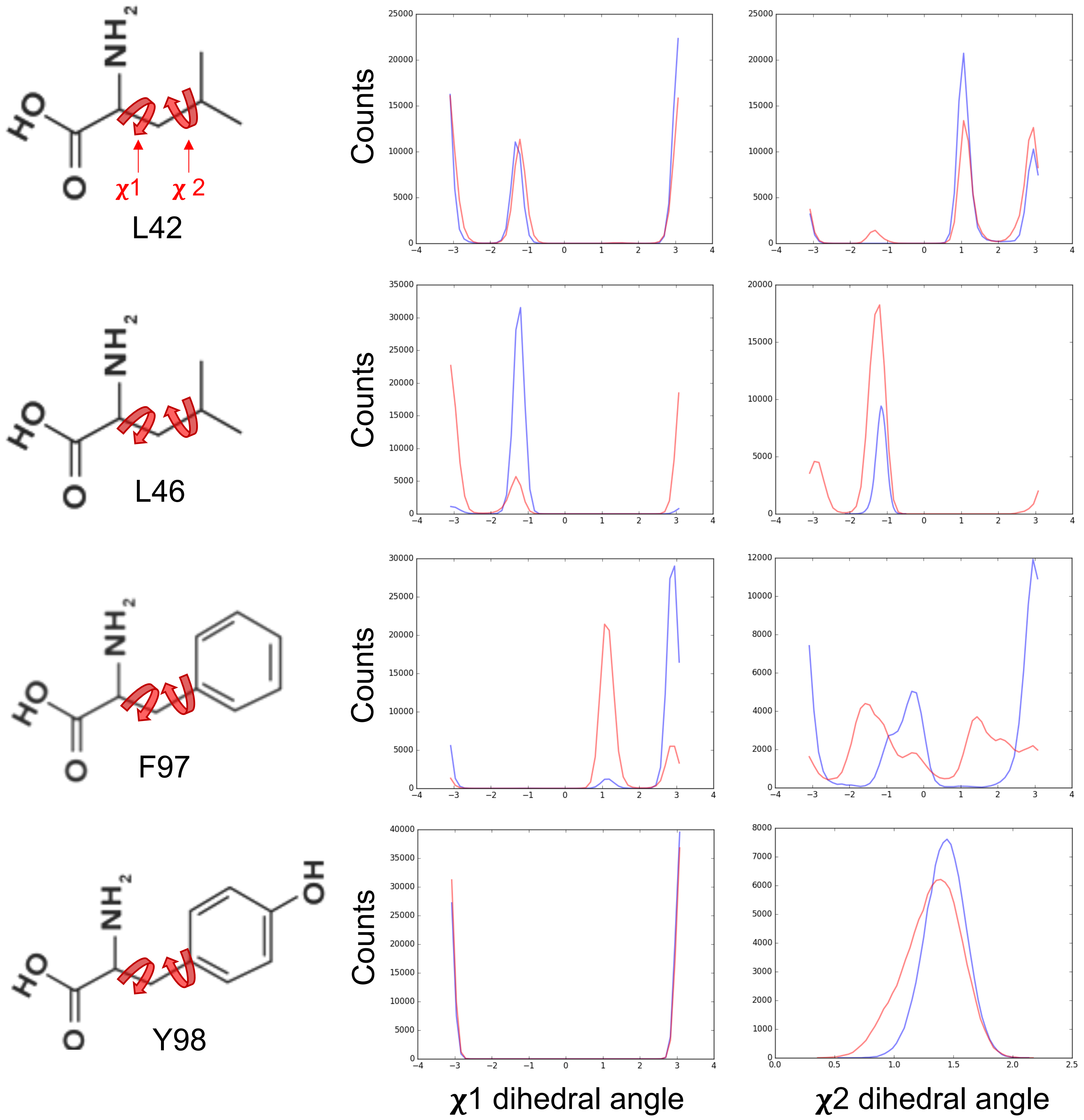
χ1
χ 2
L42
L46
F97
Y98
Counts
Counts
Counts
Counts
χ1 dihedral angle
χ2 dihedral angle

Fig. S10

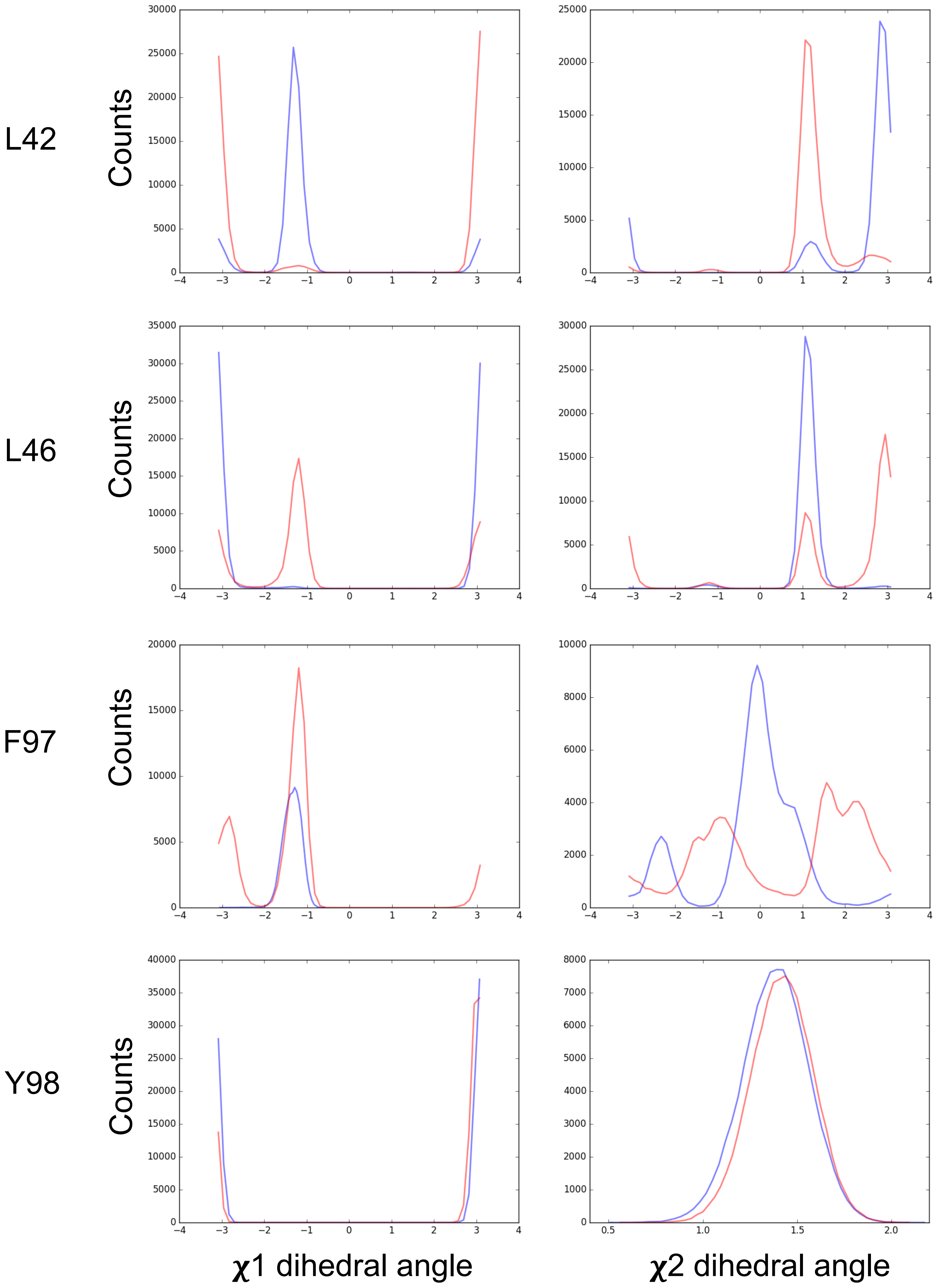

Fig. S11

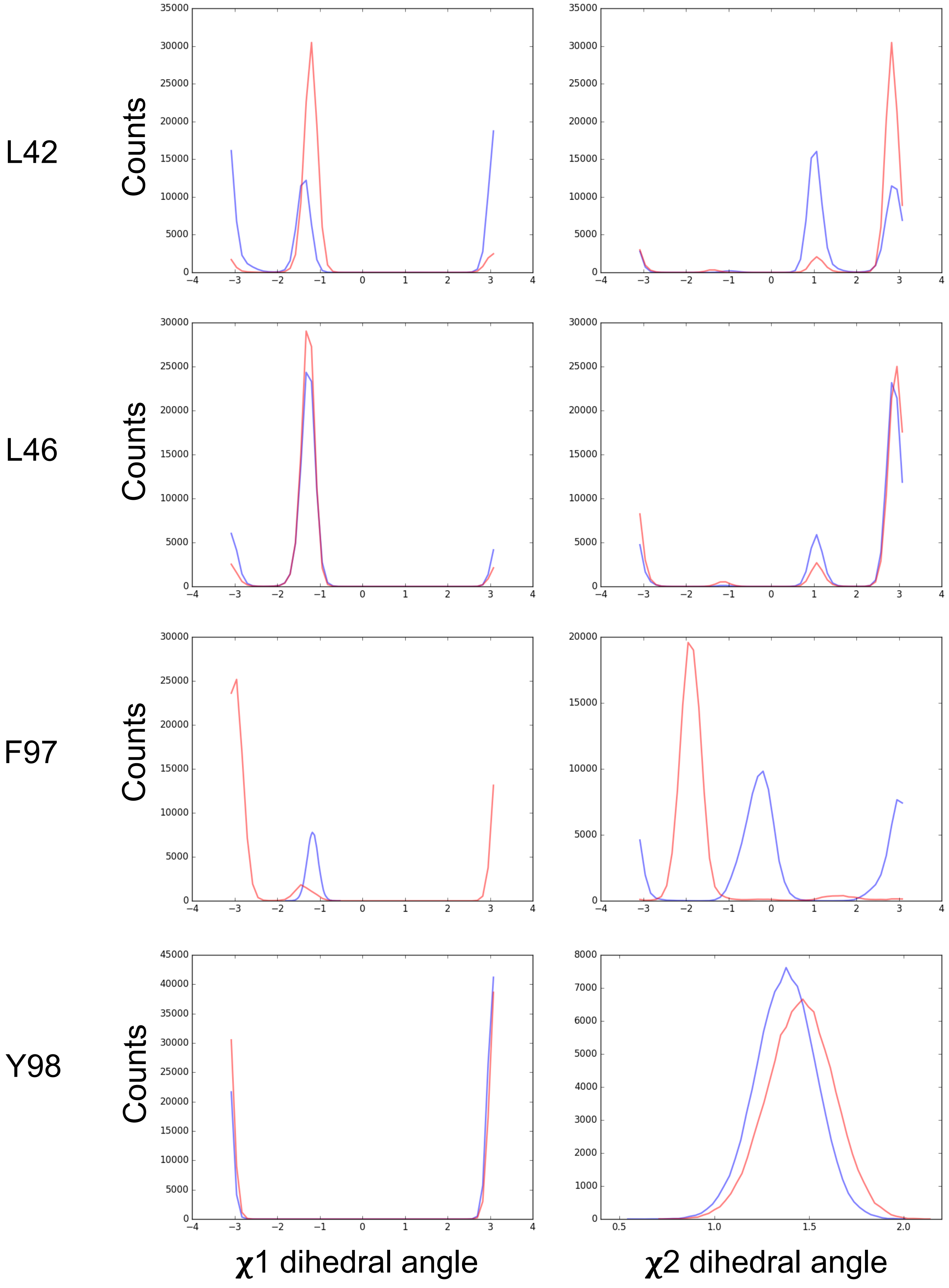

Fig. S12

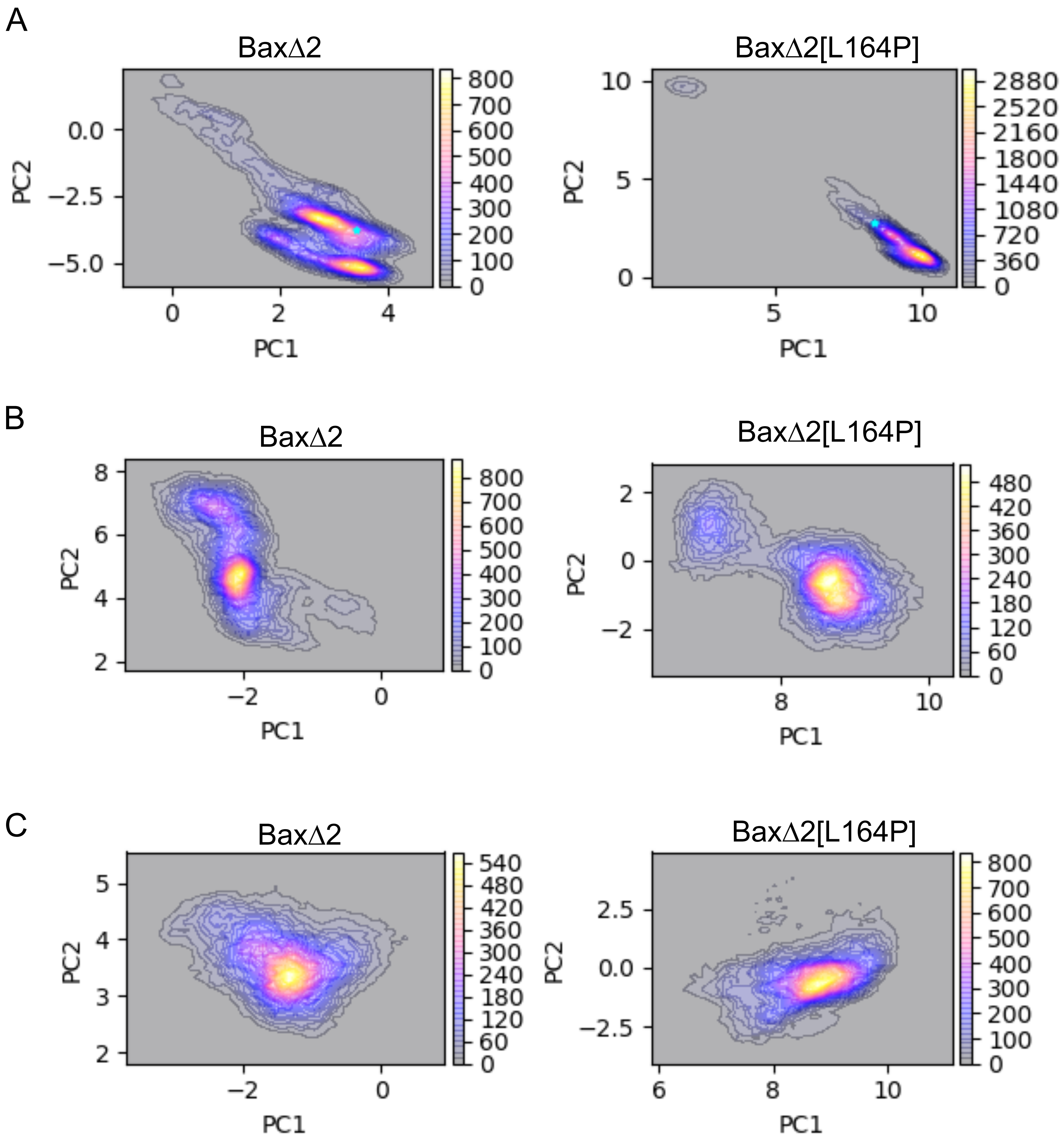

A
BaxΔ2
BaxΔ2[L164P]
B
BaxΔ2
BaxΔ2[L164P]
C
BaxΔ2
BaxΔ2[L164P]
PC1
PC2

Supplementary figure legends

Fig. S1 Alignment of predicted (blue) and crystallographic (grey) structures of Baxα. The structures were aligned using VMD 1.9.0.

Fig. S2 RMSDs of backbone atoms in the core (upper left), α1 (upper right), α2 (lower left), and α9 (lower right), relative to the initial structure, during the second repetition of 200 ns of MD simulations. Coloring is by variant: Baxα (grey), Baxα[L26P/L27P] (blue), BaxΔ2 (red), and BaxΔ2[L164P] (orange). Shaded lines show RMSDs for all recorded points and solid lines are smoothed as described in section 4.4.

Fig. S3 RMSDs of backbone atoms in the core (upper left), α1 (upper right), α2 (lower left), and α9 (lower right), relative to the initial structure, during the third repetition of 200 ns of MD simulations. Coloring is by variant: Baxα (grey), Baxα[L26P/L27P] (blue), BaxΔ2 (red), and BaxΔ2[L164P] (orange). Shaded lines show RMSDs for all recorded points and solid lines are smoothed as described in section 4.4.

Fig. S4 A dihedral plot of L25. (**A)** The dihedral plot of L25 at helix α1 kink position along the simulation trajectory. Baxα (grey), Baxα[L26P/L27P] (blue). (**B**) The dihedral plot of L25 along the second (**B**) and third (**C**) repeated simulation trajectories.

Fig. S5 (A) RMSDs of backbone atoms, relative to the initial structure, of the top and bottom half of helix α1 during 200 ns of simulation. For each snapshot, the same α carbons are used for alignment and RMSD calculation. The second repeated (**B**) and third repeated (**C**) simulations. Coloring is by variant: Baxα (grey), Baxα[L26P/L27P] (blue).

Fig. S6 RMSDs of backbone atoms, relative to the initial structure, of helix α3, α4, α5, α6, α7, α8, during 200 ns of simulation are shown. Coloring is by variant: Baxα (grey), Baxα[L26P/L27P] (blue), BaxΔ2 (red), and BaxΔ2[L164P] (orange).

Fig. S7/S8 RMSDs of backbone atoms, relative to the initial structure, of helix α3, α4, α5, α6, α7, α8, during the second repeatd (**S7**) and third repeated (**S8**) simulations are shown. Coloring is by variant: Baxα (grey), Baxα[L26P/L27P] (blue), BaxΔ2 (red), and BaxΔ2[L164P] (orange).

Fig. S9 Calculation of dihedral angles. The $\chi 1$ and $\chi 2$ of Amino acids L42, L46, F97, Y98 (from top to bottom) were analyzed by using MDTraj 1.9.3. BaxΔ2 colored as blue, BaxΔ2[L164P] colored as red.

Fig. S10/S11 Calculation of dihedral angles. The $\chi 1$ and $\chi 2$ of Amino acids L42, L46, F97, Y98 (from top to bottom) in the second (**S10**) and third (**S11**) repeated simulations were analyzed by using MDTraj 1.9.3. BaxΔ2 colored as blue, BaxΔ2[L164P] colored as red.

Fig. S12 Principal component analysis (PCA). (**A**) PCA landscapes of BaxΔ2 (left) and BaxΔ2[L164P]. The blue spot pointed at the location of BaxΔ2 or BaxΔ2[L164P] docking structure which were shown in figure 7. PC, principal component. PCA landscapes of the second (**B**) and third (**C**) repeated MD simulation.
